# Potential curing and beneficial effects of Ooitabi (*Ficus pumila* L.) on hypertension and dyslipidaemia in Okinawa

**DOI:** 10.1111/jhn.12806

**Published:** 2020-08-26

**Authors:** K. Suzuki, K. Gonda, Y. Kishimoto, Y. Katsumoto, S. Takenoshita

**Affiliations:** ^1^ Daido Obesity and Metabolism Research Center Naha Japan; ^2^ Daido Central Hospital Naha Japan; ^3^ Department of Bioregulation and Pharmacological Medicine Fukushima Medical University Fukushima Japan; ^4^ Executive Office of the Governor Okinawa Prefectural Government Naha Japan; ^5^ Faculty of Science Fukuoka University Jonan Japan; ^6^ Fukushima Medical University Fukushima Japan

**Keywords:** Ooitabi, *Ficus pumila* L, hypertension, dyslipidaemia, flavonoid

## Abstract

**Background:**

Over 30% of the population of Okinawa Prefecture have a high body mass index. The incidence of hypertension and dyslipidaemia has also increased in recent years. We found that Ooitabi (*Ficus pumila* L.), a plant native to Okinawa, was useful for hypertension. During ancient times, the extracts of Ooitabi leaves were used for making Ishimaki tea in some areas of Okinawa Prefecture. The plants in Okinawa are rich in antioxidants, and four flavonoid glycosides, including rutin, have been identified in Ooitabi.

**Methods:**

In the present study, we conducted clinical verification tests on the effects of drinking Ishimaki tea on outpatients with hypertension and dyslipidaemia. Of 3814 Japanese patients who underwent medical check‐ups in Okinawa, 38 individuals with high blood pressure, dyslipidaemia, liver dysfunction and gout visited our hospital as outpatients and were asked to drink Ishimaki tea.

**Results:**

After 3 months, there were significant reductions in body mass index, systolic and diastolic blood pressure, total cholesterol, low‐density lipoprotein cholesterol, γ‐glutamyltrans peptidase, uric acid and ratio of blood vessel insulin resistance.

**Conclusions:**

Ooitabi extract can lower blood pressure and improve lipid abnormalities and has likely contributed to the well‐known health and longevity of the population in Okinawa.

## Graphical abstract

Shisa is Okinawan lion (or lion dog) statue placed as talisman against evil at entrances, on roofs and on stone walls. Shisa holds Ooitabi leaves in its mouth (Fig. 2a).

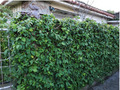

## Introduction

Of 3814 individuals who underwent health check‐ups at our hospital in Okinawa Prefecture from January 2019 to September 2019, 1432 (37.5%) had a high body mass index (BMI) (≥25 kg m^−2^), 1408 (36.9%) had high systolic blood pressure (sBP) (≥130 mmHg), 1019 (26.7%) had high diastolic blood pressure (dBP) (≥85 mmHg), 792 (27.4%) had high total cholesterol (TC) (≥220 mg dL^−1^), 1468 (46.0%) had high low‐density lipoprotein cholesterol (LDL‐C) (≥120 mg dL^−1^), 1462 (45.8%) had high γ‐glutamyl trans peptidase (γ‐GTP) (≥50 IU L^−1^), 580 (24.6%) had high uricacid (UA) (≥7.1 mg dL^−1^), 741 (23.2%) had high triglyceride (TG) (≥150 mg dL^−1^), 207 (6.4%) had low high‐density lipoprotein cholesterol (HDL‐C) (≤39 mg dL^−1^) and 695 (21.8%) had high vascular insulin resistance defined as a TG/HDL ratio >3. In Okinawa Prefecture, the percentage of people who have high BMI, high BP and dyslipidaemia has reached 30%. Despite the availability of drugs for treatment, several of these individuals have not started any pharmacotherapy. Therefore, the management of the health of such individuals is a major issue and should entail specific health check‐ups in Okinawa Prefecture and health guidance, according to the implementation plan of the Naha City Health Department Specific Health Checkup Division (i.e. Naha City second phase insurance business operation plan or data health plan). To enhance the effectiveness of the plan, Healthy Naha 21 (second phase) was designed as an insurance and welfare plan comprising a review of eating habits. Notably, the population of Okinawa Prefecture is known to have longevity and traditional eating habits ^(^ ^1^ ^)^. Okinawa is also known for its people’s long lifespans ^(^ ^2^, ^3^ ^)^. One contributing factor is the ingestion of a group of locally grown plants, called island vegetables, from ancient times onwards. As a result of its subtropical location, Okinawa enjoys a warm climate all year round. Perhaps because of this climate, the plants are extremely diverse, and there are many heteromorphic plants of primary colors, which protect them from ultraviolet radiation. The plants are known to produce phytochemicals ^(^ ^4^ ^)^, which are non‐nutritional physiologically active substances contained in plants, such as vegetables, fruits and grains, and include polyphenols, organic sulphur compounds and carotenoids. Although these plant‐derived compounds are not required to maintain physical function, they may have a positive influence on health ^(^ ^5^ ^)^.

In Okinawa, it is reported that people have been drinking the extraction of Ooitabi plant for their health for more than 500 years. However, the antihypertensive and fat‐lowering benefits of Ooitabi leaves have not been confirmed clinically. Epidemiological studies of the health relevance with respect to anti‐metabolism and Ooitabi polyphenol consumption still remain outstanding.

The present study is focused on the effects of Ooitabi leaves on hypertension and dyslipidaemia among Japanese patients who underwent medical check‐ups in Okinawa Prefecture.

## Materials and methods

### Study setting

**Figure 1:**
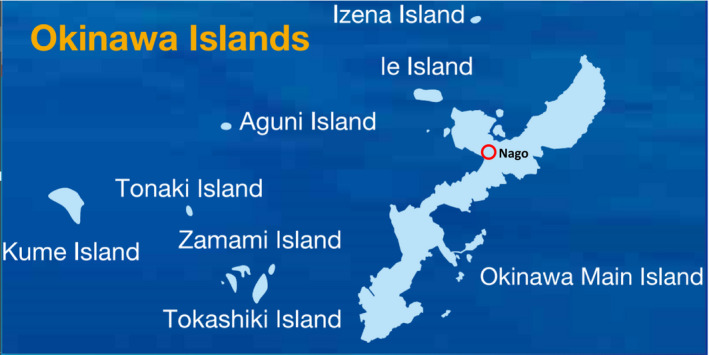
Vegetation and location of Ooitabi. Ooitabi grows in Nago City of Northern Okinawa. This is from the official homepage of the Japanese Municipality of Okinawa Prefecture (Download Brochure/Official Website of Okinawa Prefecture; outline of Okinawa Prefecture; https://www.pref.okinawa.jp/site/chijiko/kohokoryu/foreign/english/download.html.

**Figure 2:**
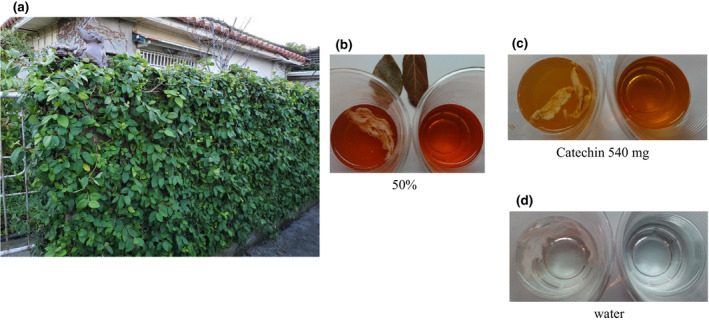
Vegetation and extraction of Ooitabi and subcutaneous fat from an Au pig. Ooitabi grows on stone walls (a). After collection, the dried Ooitabi leaves are then extracted with steeped in hot water. For 24 h, the subcutaneous fat was left in commercially available cups containing Ooitabi extracts at 50% (b), drinking water containing catechin at concentrations of 540 mg per 500 mL (c) and pure water (d). The cups on the left contain fat, whereas those on the right contain the reference solution without fat. (b–d). An Ooitabi leaf after extraction. The cups with Ooitabi extract (b) and catechin (c) have almost similar levels of dissolved fat.

From ancient times onwards, elderly individuals with high BP living in the northern part of the Okinawa Main Island (Figure 1) have been drinking Ishimaki tea, which was made by drying the stems and leaves of Ooitabi, followed by extraction of the major components with water. Ishimaki tea is said to have antihypertensive effects, and its name is derived from the fact that Ooitabi winds up (maki‐tsuku) on stone walls (Ishi‐gaki). An Ooitabi plant winding up stone walls is shown in Figure 2a; its leaves are green but turn white after drying for 1 day. When 20–25 g of the dried leaves are placed in 1 L of boiling water at 100 °C for 7 min, a brown liquid is produced (Figure 2b), which is mixed with a flavour and heated for approximately 2 min to yield the more palatable Ishimaki tea compared to the original extract.

### Participants and study design

Among the 3814 Japanese patients who underwent medical check‐ups in Okinawa Prefecture, volunteers (*n* = 38) with upper borderline high BP and dyslipidaemia were asked to drink approximately 200–300 mL of Ishimaki tea a day. These volunteers did not seek treatment on their own but were outpatients who were undergoing periodic medical examinations, comprising blood collection and follow‐up. Moreover, these outpatients had never consumed Ishimaki tea or had taken consumed it in the remote past. After 3 months of drinking Ishimaki tea, the participants were re‐evaluated with respect to BMI and hypertension, and underwent blood sampling for measurement of TC, LDL‐C, γ‐GTP, UA, TG and HDL‐C levels. All participants provide their written informed consent forms to disclose the results and study details in scientific articles.

### Source of plants and plant specimens

*Ficus pumila* was introduced to western science in 1721. It was first described and named as *Ficus stipulata* in 1753 by Carl Linnaeus. The species name ‘pumila’ is a derivation of the Latin word for ‘dwarf’. This refers to the juvenile growth, which bears small, ovate or heart‐shaped leaves of no more than 2.5 cm ^(^ ^6^ ^)^. There are more than 850 members of the *Ficus* genus; among them are several species commonly available for indoor gardeners, including the creeping fig, or *F. pumila* (sometimes also called *Ficus repens*) ^(^ ^7^ ^)^. The *Ficus* genus contains some of the most beautiful, widespread and important plants in the world. The typification of the name *Ficus pumila* var. *pumila* (*Moraceae*) is discussed. The designation of the corresponding type is based on the consultation of Linnaeus’s original material and the literature cited in the protologue ^(^ ^8^ ^)^. Similar to other plant species in the family Moraceae, contact with the milky sap of *Ficus pumila* can cause phytophotodermatitis, a potentially serious skin inflammation ^(^ ^9^ ^)^.

However, stems and leaves of *Ficus pumila* L. have been used in Traditional Chinese Medicine as a tonic and to treat fever. Leaves of *Ficus pumila* L. are used in Japan in beverages to treat diabetes and high blood pressure ^(^ ^10^ ^)^. *Ficus pumila* L. is native to East Asia, and also inhabits warm areas, such as Okinawa. *Ficus pumila* L. has been known as ‘Ooitabi’ in Okinawa from ancient time onwards.

These plants have been reported to have antioxidant activity ^(^ ^11^ ^)^. As shown in Figure 2a, the plant (Ooitabi) grows on stone walls of any civilian houses of Nago area (i.e. Nago City) (Figure 1). Thus, seeds were not obtained commercially, nor were they acquired from another laboratory. Because these samples(plants) were obtained from the stone walls of civilian houses (from the wild), special permission was not necessary to collect the samples (other than thanking the house owners). Because plants (Ooitabi) on stone walls have been collected for a long time by civilians for making tea, there are institutional or national guidelines regarding the usage of such plants. Therefore, special permissions and/or licenses are not necessary.

### Description of materials

The ripe fruit pod taken from the female strain of Ooitabi has a sweet and mellow taste and has been said to be sweeter than figs. Ooitabi fruits have been shown to suppress nitric oxide production in diabetes and cancer and to exert an antioxidant action in the 2,2‐diphenyl‐1‐picryl hydrazyl radical scavenging effect test ^(^ ^12^ ^)^. However, eating the fruit does not appear to be a common practice. On the other hand, Ooitabi stems and leaves have been widely consumed for tonicity, common cold and diabetes. Four types of flavonoids, including apigenin 6‐neohesperidosy, kaempferol 3‐robinobioside, kaempferol 3‐rutinoside ^(^ ^13^, ^14^, ^15^, ^16^, ^17^ ^)^ and rutin, can be extracted from Ooitabi leaves and had been shown to have antioxidant actions and may further improve diabetes ^(^ ^18^ ^)^.

The study was reviewed and approved by the Daido Central Hospital. IRB and informed consent were obtained from each subject prior to their participation in the study. Statistical analysis was only conducted if the variation with a treatment (SD divided by the means) was greater than 10% and the difference among treatment means was less than 3 SDs.

## Results

### The clinical effect of Ishimaki tea

**Figure 3:**
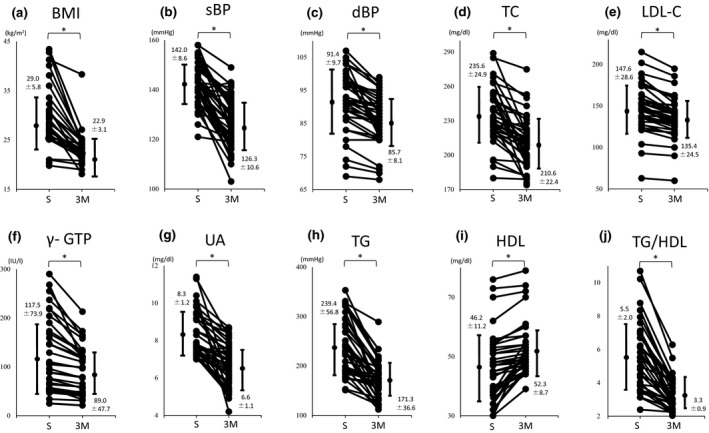
Results after drinking Ooitabi extracts. BMI (a), sBP (b), dBP (c), TC (d), LDL‐C (e), γ‐ GTP (f), UA (g), TG (h) and TG/HDL (j) significantly decreased. HDL‐C (i) increased. S, start date; 3M, After 3 months; sBP, systolic blood pressure; dBP, diastolic blood pressure; TC, total cholesterol; LDL‐C, low‐density lipoprotein cholesterol; γ‐GTP, γ‐glutamyl trans peptidase; UA, uric acid; TG, triglyceride; HDL‐C, high‐density lipoprotein cholesterol; TG/HDL, triglyceride to high‐density lipoprotein cholesterol ratio. Data are presented as the mean (SD). *P* values were determined using Student's *t* test.

As shown in Figure 3, drinking Ishimaki tea for 3 month significantly reduced mean (SD) body mass index [29.0 (5.8) versus 22.9 (3.1) mmHg, *P* < 0.001]; systolic BP [142.0 (8.6) versus 126.3 (10.6) mmHg, *P* < 0.001]; diastolic BP [91.4 (9.7) versus 85.7 (8.1) mmHg, *P* = 0.007]; TC [235.0 (24.9) versus 210.6 (22.4) mg dL^−1^, *P* < 0.001]; LDL‐C [147.6 (28.6) versus 135.4 (24.5) mg dL^−1^, *P* = 0.048]; γ‐GTP [117.5 (73.9) versus 89.0 (47.7) mg dL^−1^, *P* = 0.05]; UA [8.3 (1.2) versus 6.6 (1.1) mg dL^−1^, *P* < 0.001]; TG [239.4 (56.8) versus 171.3 (36.6) mg dL^−1^, *P* < 0.001]; and TG/HDL‐C [5.5 (2.0) versus 3.3 (0.9) mg dL^−1^, *P* < 0.001], whereas HDL was significantly increased [46.2 (11.2) versus 52.3 (8.7) mg dL^−1^, *P* = 0.011].

### The biological effect of Ishimaki tea

In another experiment (Figure 2), the subcutaneous fat of an Agu pig from Okinawa Prefecture was immersed in either Ooitabi extracts, catechin or pure water, followed by observation of the degree of turbidity over time. In the liquid containing the Ooitabi extract, a significant portion of the fat dissolved and the liquid became turbid after 24 h. The liquid with 50% Ooitabi extract yielded almost the same turbidity as that of the liquid containing 540 mg per 500 mL of catechin. The extracted (100%) Ooitabi liquid was very opaque and cloudy, precluding any comparison of its gross and photographic appearance with those of the other stock solutions.

## Discussion

Among these health check‐up patients with hypertension, 44% appeared to be have hereditary hypertension. In particular, some cases with a family history of hypertension had already acquired high BP by the age of 40 years. One 23‐year‐old man who had hypertension had a family history of the condition in his grandparents and parents. Although the number of cases was small, Ooitabi extracts have the potential to prevent hypertension, even the familial type. Future verification is necessary to confirm this.

Although not very significant, intake of Ooitabi extracts effected some improvements in dyslipidaemia. The 38 participants with dyslipidaemia in the present study found it difficult to take medication, practice good nutritional intake and exercise. Fortunately, drinking Ishimaki tea clearly lowered their cholesterol levels. Although the mechanism is unknown, the antioxidant action of the Ooitabi extracts might have contributed. Notably, in these cases, the reductions in LDL‐C and γ‐GTP were minimal. However, the significant increase in HDL‐C likely reduced insulin vascular resistance ^(^ ^19^ ^)^. These findings implied that drinking Ishimaki tea may prevent arteriosclerosis and decrease the risk for diabetes. It was also considered to be useful for the increasing number of dialysis patients in Okinawa.

To convince people to drink Ishimaki tea in their daily life, the degree of fat dissolution was evaluated in a simple cup and was made easy to understand. After soaking for 24 h, the Agu pig fat dissolved in the liquid containing the Ooitabi extracts, making the liquid appear turbid. The degree of turbidity was the same between a 50% concentration of Ooitabi extracts and a catechin‐containing tea, which is sold in the market at a concentration of 540 mg per 500 mL. When immersed in pure water, the fat did not dissolve and the water did not become turbid.

Although the degree of turbidity was not quantified, Okinawa people usually drink Ishimaki tea with a 50% Ooitabi extract concentration. *Ficus pumila* L. contains flavonoids such as apigenin, kaempferol and rutin. There are some reports that flavonoids regulate lipolysis. Flavonoids constitute a major group of polyphenolic compounds that are directly associated with the organoleptic and health‐promoting properties of red wine. The way that wine flavonoids may be absorbed and metabolised could interfere with their bioavailability and therefore in their health‐promoting effect ^(^ ^20^ ^)^. Black tea polyphenols exert a positive effect with respect to inhibiting obesity via major mechanisms: promoting lipid metabolism by activating AMP‐activated protein kinase to attenuate lipogenesis and enhance lipolysis, and decreasing lipid accumulation by inhibiting the differentiation and proliferation of preadipocytes ^(^ ^21^ ^)^. The mechanisms involved in weight loss in which polyphenols may have a role are: activating β‐oxidation; a prebiotic effect for gut microbiota; inducing satiety; stimulating energy expenditure by inducing thermogenesis in brown adipose tissue; modulating adipose tissue inhibiting adipocyte differentiation; and promoting adipocyte apoptosis and increasing lipolysis ^(^ ^22^ ^)^. It was reported that almond skin polyphenol extract significantly promoted phosphorylation of AMP‐activated protein kinase, increased the activity of adipose triglyceride lipase and hormone‐sensitive lipase, inhibited adipogenesis‐related transcription factors, and regulated lipolysis ^(^ ^23^ ^)^.

In conclusion, a possible reason for the longevity exhibited by those living in Okinawa Prefecture has been reported ^(^^24^^)^. Ooitabi is a wild plant that is native to Okinawa, although it is difficult to cultivate, being time consuming and labor intensive, with low‐volume productivity and an unpalatable taste. Therefore, not all Okinawa people drink Ishimaki tea. Nevertheless, Ooitabi has possibly contributed to longevity from ancient times onwards. Therefore, the tradition or old practice of drinking Ishimaki tea should be preserved and carried on, aiming to protect health and achieve a longevity of over 100 years old not only for the population of Okinawa and Japan, but also for the general population worldwide, where the incidence of high blood pressure and dyslipidaemia, as well as diabetes and hyperuricemia, has been increasing.

### Study strengths and limitations

There is still a great difference between Ooitabi flavonoid bioavailability and their health‐promoting effects. More *in vivo* studies focused on flavonoid metabolites are still required. From this verification alone, whereas drinking Ishimaki tea apppears to reduce the amounts of subcutaneous and visceral fat tissues. The present study had some limitations. The absorption rates of the ingested components of the Ishimaki tea (i.e. Ooitabi extracts) were not measured. Therefore, the mechanisms of action off lavonoids and rutin on blood pressure and dyslipidaemia need to be clarified in future studies. Moreover, the quality and quantity of each Ooitabi extract component was not clear. Nevertheless, the appropriate extraction time and concentration were followed, according to the methods used from ancient times onwards in Okinawa. Although the number of participants was small, the present study clearly showed that the Ooitabi extracts lowered the blood pressure of health check‐up patients who had borderline hypertension and dyslipidaemia but in whom medical treatments are not yet indicated according to the hypertension guidelines.

## Conclusions

Drinking Ishimaki tea clearly lowered BMI, sBP, dBP, TC, LDL‐C, γ‐GTP, UA, TG, HDL‐C and TG/HDL serum levels and increased HDL‐C serum levels. The antioxidant action of the Ooitabi extracts might play an important role in the development of blood pressure, fat metabolism and gout. The effect of Ooitabi on metabolic syndrome strongly depends on the extents of high BP, hyperlipidaemia and hyperuricemia that occur with the life style disease.

## Transparency declaration

The lead author affirms that this manuscript is an honest, accurate and transparent account of the study being reported. The reporting of this work is compliant with STROBE guidelines. The lead author affirms that no important aspects of the study have been omitted and that any discrepancies from the study as planned have been explained.
